# Pennsylvania Hospital

**Published:** 1859-03

**Authors:** 


Pennsylvania Hospital.—We regret to announce the resignation of Dr. William Pepper, of the post of Physician to this institution, which he has held for many years to the perfect satisfaction of every one connected with it, and those seeking the benefits of his instructions. As yet, the vacancy has not been filled.

